# Dietary constraints can preclude the expression of an honest chemical sexual signal

**DOI:** 10.1038/s41598-017-06323-8

**Published:** 2017-07-20

**Authors:** Roberto García-Roa, Jorge Sáiz, Belén Gómara, Pilar López, José Martín

**Affiliations:** 1 Department of Evolutionary Ecology, National Museum of Natural Sciences, Spanish Research Council (MNCN- CSIC), José Gutiérrez Abascal, 2, 28006 Madrid, Spain; 2 Department of Instrumental Analysis and Environmental Chemistry, Spanish Research Council (IQOG- CSIC), Juan de la Cierva, 3, 28006 Madrid, Spain

## Abstract

Identifying the factors that underlie signal divergences remains challenging in studies of animal communication. Regarding the chemical signalling, different compounds can be found in some species but be absent in others. We hypothesized that if the costs that are associated with the expression of some compounds are too high, their presence in the signal may be restricted. However, these compounds may be expressed and be functional when those costs are relaxed. Vitamin E (α-tocopherol), a dietary compound with metabolic relevancy, acts as an honest chemical sexual signal in many lizards but no in others such as the Carpetan Rock lizard (*Iberolacerta cyreni*). We investigated whether dietary supplementation favours the expression of this vitamin in scents of *I. cyreni*. We show that dietary constraints can preclude the expression of vitamin E in chemical secretions of wild males because was expressed when it was experimentally provided in the diet. Vitamin E supplementation also heightened the immune response of males and increased the interest of their scent for females, highlighting the vitamin E as a chemical sexual signal in this species. We suggest that diet could decisively act as a driver of intra- and interspecific divergences in the chemical signalling of lizards.

## Introduction

The ‘handicap paradigm’ proposes that individuals that are able to afford the costs associated with the elaboration and maintenance of secondary sexual characters are favoured by selection because these individuals display honest signals that truly convey useful information on the quality of bearers to the receivers (competitors and potential mates)^1, 2^. In this context, the way in which ecological factors contribute to the expression of these honest signals is a subject of continued research. However, despite the fundamental role that chemical signalling plays in sexual selection and the diversification of many species^3, 4^, this question has mainly been examined in visual traits^5, 6^.

The foregoing reviews highlight lizards and their chemical signalling as an insightful model system to improve the knowledge on social and sexual animal interactions^7–9^ because the different structures (*i.e*., composition) of scents usually determines the response of receivers and also the reproductive and survivorship success of the emitter (see examples in ref. 8). Most of the evidence in this regard comes from studies that analyse the scents produced by femoral and precloacal glands, typically denoted as ‘chemical secretions’^10, 11^. The qualitative and quantitative composition of the lipophilic fraction of these chemical secretions differ among individuals, populations and species^12–14^, and this variation is used by lizards in social and sexual interactions to assess individual differences in many traits^15–17^. The composition of the chemical secretions seems to be highly dependent on physiological processes and endocrine regulation^8^ that occur while individuals are under biotic (*e.g*., trophic resources^18^) and abiotic (*e.g*., climatic conditions^19^) pressures. However, identifying which factors underlie the intra- and interspecific divergences in chemical signalling is still a pending task.

In this regard, diet is a major driver of differences in sexual ornamentation because diet is particularly important in the chemical signalling of many organisms^18^. However, it is still unclear how diet influences the expression of honest chemical signals in lizards. Several compounds with a dietary origin that can act as potentially honest chemical signals, such as vitamin E (α-tocopherol; VE), have been described in the chemical secretions of many lizard species but not in others^10^. This vitamin is only synthesised by plants or microorganisms and must be acquired by animals through their diet^9^. VE is a radical scavenger and antioxidant involved in cell membrane defence and with relevant roles in the immune response^20, 21^. VE deficiency produces neurological disorders and physiological diseases^22, 23^. However, although VE participates in many metabolic and physiological pathways, lizards of some species allocate large amounts of this vitamin in chemical secretions that are deposited outside of the body. The magnitude of such allocation has been correlated with the quality of males in some species, which may explain the preference of females for the scent of those males^24–26^. Only best-quality males can obtain enough VE in their diet to support physiological functions (for example, to maintain and enhance the immune response^24, 26^) and, at the same time, allocate high levels of VE to chemical signals. Nevertheless, in addition to a signalling function of male quality, a pre-existing sensory bias in females for a chemosensory search of VE in their food might simply explain why females also preferred chemical secretions of males with higher proportions of VE^27, 28^.

The Carpetan rock lizard (*Iberolacerta cyreni*) is a species often used as a model to study chemical ecology in lizards (see refs 7, 9, 11). Although previous analyses have not identified VE in chemical secretions of *I. cyreni* ^16^ nor in phylogenetically related species such as *I. monticola* and *I. galani* ^29, 30^, recent research revealed that other closely related species within the same genus (*i.e*., *I. aurelioi, I. bonnali* and *I. martinezricae*) divert large amounts of VE to chemical secretions^30^. In the present study, we investigated whether a low availability in the diet of VE explained the lack of expression of this vitamin as a chemical signal in male *I. cyreni*. We experimentally supplemented male lizards with VE in their diet and examined changes in the composition of chemical signals. We hypothesized that the allocation of a particular compound (*e.g*., VE) in the chemical signal could be restricted if this compound was scarce and the cost associated with its expression, such as the diversion from important metabolic functions, was too high. However, if the costs were relaxed (*e.g*., when the availability of VE in the diet increased) those compounds could be expressed and retained or acquired a signalling function. Thus, to further assess whether this potential signal might convey honest information, we examined the relationship between signal composition and immunity, as well as the chemosensory responses of females to the scent of males as a measure of the females’ interest.

## Results

### Analysis of chemical secretions

**Figure 1:**
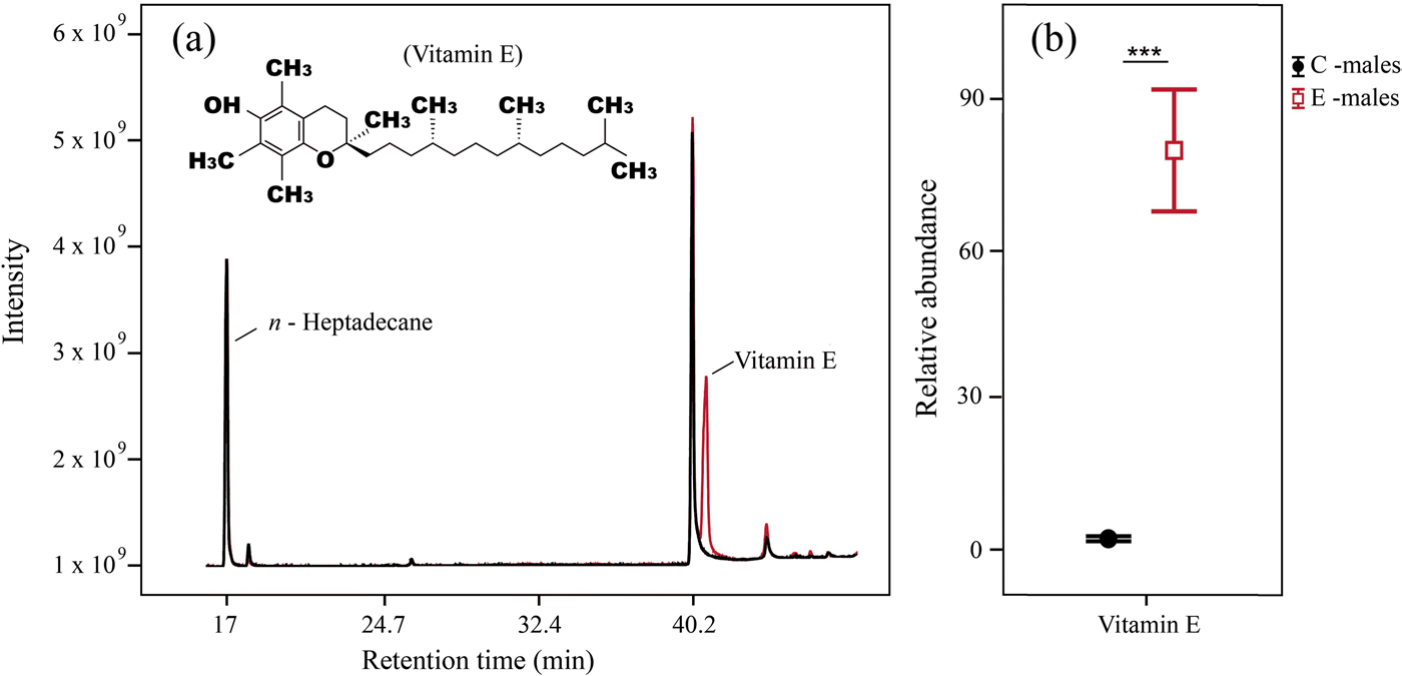
Chromatograms from femoral secretions of *Iberolacerta cyreni* male lizards. (**a**) Comparison of chromatograms from two different treatments: ‘C-males’ (black) were supplemented with soybean oil, and ‘E-males’ (red) were supplemented with vitamin E. Peaks of interest in this study are the *n*-heptadecane (internal standard; IS) and vitamin E (α-tocopherol), which is especially visible in E-males. (**b**) Comparison of the relative abundances (mean ± SE) of vitamin E in secretions of C-males and E-males.

The chemical analyses revealed the presence of VE in secretions of *I. cyreni* males at the end of the dietary supplementation (Fig. 1a). In control males (C-males), the main classes of compounds found in secretions were steroids (91.76% of TIC), followed by carboxylic acids (2.56%), alcohols (1.67%), aldehydes (1.45%), waxy esters (1.23%), VE (1.21%) and squalene (0.12%). In vitamin E dietary supplemented males (E-males), we also found steroids as the main compounds (69% of TIC). However, we subsequently found VE (28.12%) as the second most abundant compound, followed by waxy esters (0.88%), aldehydes (0.81%), alcohols (0.80%), carboxylic acids and their esters (0.32%) and squalene (0.06%). A significant decrease in the relative TIC proportions of carboxylic acids and their esters (F_1,36_ = 5.33, *P* = 0.02) and squalene (F_1,36_ = 5.37, *P* = 0.02) was detected in supplemented E-males, whereas the proportion of VE was significantly higher in secretions of E-males than in C-males (F_1,36_ = 77.78, *P* < 0.001). However, we did not find significant differences between treatments in proportions of steroids (F_1,36_ = 1.04, *P* = 0.31), alcohols (F_1,36_ = 3.94, *P* = 0.054), waxy esters (F_1,36_ = 0.51, *P* = 0.47) or aldehydes (F_1,36_ = 1.68, *P* = 0.20).

Our analyses, using the internal standard to explore potential differences in the relative abundance of VE, confirmed that E-males secreted significantly higher amounts of VE than C-males (79.42 ± 18.22 *vs*. 1.64 ± 0.36 respectively; F_1,36_ = 236.45, *P* < 0.001) (Fig. 1b). We also found small amounts of VE in C-males (1.21%), probably due to the VE traces contained in the oil used as a control for supplying these males.

### Relationships between chemical signalling and the immune response

**Figure 2:**
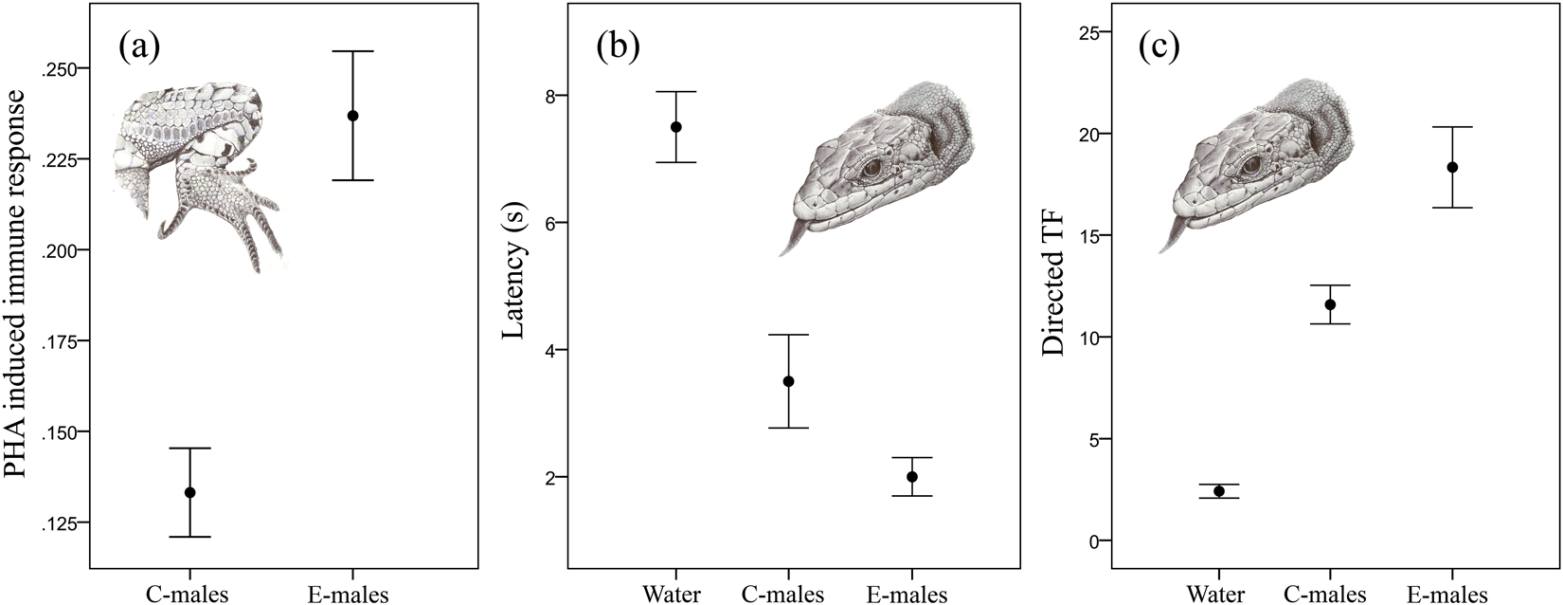
Effect of vitamin E dietary supplementation on the immune response of *Iberolacerta cyreni* male lizards and chemosensory responses of females to scent of males. (**a**) Immune response measured in control (C-males) and supplemented (E-males) male lizards. (**b**) Latencies (mean ± SE in s) and (**c**) number (mean ± SE) of directed tongue-flicks (TF) elicited by female lizards to swabs with deionised water and scents from femoral secretions of C-males and E-males.

Vitamin E supplementation affected the immune response of lizards, with E-males having significantly higher immune responses than C-males (One-way ANOVA; F_1,36_ = 23.14, *P* < 0.001) (Fig. 2a). However, within each group of males, there were no significant relationships between the relative abundance of VE and the immune response of each individual (C-males: r^2^ = 0.05, F_1,18_ = 0.084, *P* = 0.77; E-males: r^2^ = 0.03, F_1,18_ = 0.45, *P* = 0.50).

### Chemosensory responses of females to the scent of males

In all of the cases, the females responded to the impregnated swabs by tongue flicking (TF). Our results showed significant differences in latency among scent stimuli (repeated measures ANOVA; F_2,22_ = 20.77, *P* < 0.001) (Fig. 2b). Responses to swabs with water had significantly longer latencies than those to swabs with males’ scent (Tukey´s test: *P* < 0.0001 in both cases), but there were no significant differences between latencies to secretions of E-males and C-males (*P* = 0.11). The overall rate of TF to swabs significantly differed among the types of stimuli (repeated measures ANOVA; F_2,22_ = 118.13, *P* < 0.001) (Fig. 2c). Swabs with water triggered significantly lower TF rates than those with secretions of any male (Tukey´s test: *P* < 0.0001 in both cases). Moreover, swabs with secretions of E-males received significantly higher TF responses than swabs with secretions of C-males (*P* = 0.01).

## Discussion

The present study showed that the expression of an honest chemical sexual signal could be significantly affected by its dietary availability to the point of precluding its entire expression in all of the individuals of a population or species, which probably explained the absence of VE in femoral secretions of wild *I. cyreni* ^10, 16^. However, our experiment also revealed that increasing the availability of VE in the diet drove metabolic changes that enhanced the immune responses and allowed the expression of this vitamin in chemical secretions of *I. cyreni*. The dietary supplementation modified the composition of male chemical secretions, with a notable increase in VE, which probably explained why females showed a higher chemosensory response to chemical secretions of supplemented males, which could indicate a higher interest of females for the scent of these males^9, 10^.

The role of some chemical signals in social and sexual behaviour has been supported for several lizard species, especially for those signals believed to be honest, such as VE^23–26^. However, the reason why these compounds have been found in some species and not in others has been unclear. In this experiment, we detected VE in chemical secretions of *I. cyreni* for the first time, but only after males received a dietary supplementation of this vitamin. Therefore, a dietary constraint in the natural populations could explain why this vitamin had not been detected before in wild individuals^10, 16^. The absence of particular compounds in chemical secretions of some lizard species should not lead to the conclusion that these species are unable to express these compounds. As in the case of *I. cyreni* and VE, striking divergences in chemical profiles of scents might be unleashed by ecological factors rather than phylogenetic differences *per se*. Namely, due to the antioxidant and immunostimulatory properties of VE^24, 31^, lizards must trade-off between the associated costs and benefits of diverting this vitamin from metabolism to chemical secretions. The result of such trade-off is indicative of male quality^25, 26, 31^. In addition, lizards mainly thermoregulate by exposing themselves to solar radiation, whose ultraviolet fraction has oxidative effects. At high elevations, where thermal energy availability is low, lizards increase their basking activity^32^. Therefore, these lizards would need high amounts of this antioxidant vitamin in metabolism pathways^21, 33^, which might force many species such as *I. cyreni* to reduce VE in chemical secretions. The VE supplementation could have balanced this physiological trade-off, allowing individuals to allocate higher amounts of VE in chemical secretions without detrimental effects for their metabolism. This hypothesis may also be applicable to closely related species (*i.e*., *I. galani* and *I. monticola*) in which VE has not been found in chemical secretions. However, *Iberolacerta* lizard species from the Pyrenees (*I. aranica*, *I. aurelioi* and *I. bonnali*) do notably show this vitamin in their chemical secretions. This fact might be partially explained by interspecific potential differences in VE availability in their diets. In this respect, further research is needed to establish the relative role of biotic (*e.g*., diet, parasitism, predation) and abiotic (*e.g*., climate, microhabitat) factors in determining VE secretion patterns among species.

The levels of secretion of VE in chemical signals are determinant in intersexual interactions of several lizard species because such levels are correlated with male quality^8^. Our study supports the hypothesis that a higher availability of VE in the diet allows individuals to divert more amounts of this vitamin both for metabolism processes, which in turn increases the immune response, and for chemical signalling. Interestingly, although VE has not been previously detected in scents of wild male *I. cyreni*, females elicited higher TF rates to secretions of supplemented males. Although the proportion of other compounds (fatty acids and squalene) also changed after the supplementation, the already low proportions of these compounds decreased even more. Thus, it is unlikely that the enhanced responses of females were explained by compounds that decreased slightly in relative abundance, leaving the high proportions of VE as the most likely candidate to explain the increased chemosensory responses. This hypothesis is in line with the hypothesis that VE may function as an honest chemical sexual signal^24–26^ in this species too. Nevertheless, because VE is needed in metabolism and must be acquired from dietary sources, female preferences could be sensory biased. Similarly, behavioural experiments performed with this species showed that hungry females increased chemosensory responses to stimuli from both invertebrate prey and femoral secretions of males, suggesting that cholesta-5,7-dien-3-ol (provitamin D_3_) may be one of the compounds potentially responsible for eliciting these higher chemosensory responses^34^.

We conclude that if the costs associated with the allocation of some specific compounds in a chemical signal are too high, their presence in the signal could be restricted or even precluded entirely. Dietary constraints can contribute to increasing these costs. However, when those costs are relaxed, the compound may be expressed in the scent and even function as an honest sexual signal.

## Material and Methods

### Field work, lizard care and maintenance

The Carpetan rock lizard (*I. cyreni*) is a medium sized diurnal lacertid lizard that inhabits rocky highlands (above 1700 m elevation) along the Sistema Central Spanish Mountains, where this species is endemic. We collected live adult male (n = 38) and female (n = 13) lizards at ‘Alto del Telégrafo’ (40° 47ʹ N, 04° 00ʹ W, Sierra de Guadarrama, Madrid, Spain) during the first week of May 2015. Capture of lizards was carried out by noosing. Then, we transferred lizards to “El Ventorrillo” field station of the Museo Nacional de Ciencias Naturales (5 km from the capture area). During the study, we individually housed lizards in outdoor 51 × 36 × 28 cm PVC terraria with coconut fibre as substratum, rocks for cover and water *ad libitum*. We fed lizards with mealworm larvae (*Tenebrio molitor*) and house crickets (*Acheta domesticus*) dusted with calcium powder. We released all animals at their capture sites at the end of the experiment (approx. five weeks after capture). All experimental methods were performed in concordance of the Environmental Agency of Madrid Government (“Consejería de Medio Ambiente de la Comunidad de Madrid”, Spain), being reviewed and approved by the Animal Ethics Committee of the Museo Nacional de Ciencias Naturales (CSIC).

### Vitamin E supplementation

We randomly assigned males to two treatments, C-males (n = 19) and E-males (n = 19). Males in both treatments were of similar body size (snout-to-vent length, mean ± SE, C-males: 66 ± 1 mm, E-males: 67 ± 1 mm; One-way ANOVA; F_1,36_ = 1.23, *P* = 0.27). We administered to E-males a dietary dose of 5 μL of vitamin E supplement (synthetic (±)-α-tocopherol; purchased from Sigma-Aldrich Chemicals Co.) every two days, during a period of 30 days. The vitamin supplement mainly contained synthetic vitamin E (97%; approx. 1014 IU mL^−1^) and soybean oil (3%; approx. 0.32 IU mL^−1^ of natural vitamin E, *i.e*., D-α-tocopherol). Therefore, we provided E-males with approximately 5.05 IU of vitamin E per dose, which is below the tolerable levels of ingestion^35, 36^. C-males were provided with 5 μL of soybean oil alone, once every two days, during an interval of 30 days. We used sterile plastic syringes with a cannula to slowly deliver the solution into the mouth of lizards to ensure that all of the individuals received the entire dose. After the supplementation period, the femoral gland secretions of each male were collected and preserved at −20 °C in glass vials closed with Teflon-lined stoppers. We also obtained blank control vials using the same procedure without collecting secretions. We conducted with these blank vials the same collection and analytical methodology to compare with vials that carried secretions to exclude potential contaminants.

### Chemical analyses of chemical secretions

The secretion of each male was weighed using a XP2U ultra-microbalance (±0.1 μg) in a room at controlled temperature (20 °C). All of the laboratory supplies used for weighing were cleaned with *n*-hexane (95%) before and after the weighing of the secretions. We prepared a solution of 5 ppm of *n*-heptadecane in *n*-hexane to be used as an internal standard. Then, 1 μL of this solution was added per 20 μg of femoral secretion, and the mixture was preserved again in a new glass vial. The mixture was vortex-mixed for two minutes and left in the fridge for the precipitation of solid particles for five minutes. Subsequently, the liquid phase was collected and transferred to a total recovery glass vial. The final samples were kept at −20 °C until further analysis.

For sample extract analyses, we used a TRACE GC Ultra gas chromatograph coupled to a TSQ Quantum XLS mass spectrometer settled by a triple quadrupole analyser (Thermo Fisher Scientific Inc., Bremen, Germany) that operated in electron ionisation (EI, −70 eV of electron energy) and scan detection mode. We set the current of the filament to 150 μA. Then, we injected 2 μL of each sample extract in the gas chromatograph using a programmed temperature vaporization (PTV) injector in splitless mode. We used a split flow of 10 mL/min of helium and 2 min of splitless time. In addition, we kept the injector at 250 °C during the transfer phases with a constant septum purge. We also performed a cleaning phase of the injector after the transfer phase, increasing the injector temperature at 14.5 °C/s up to 350 °C and holding at 350 °C for 5 min. Additionally, the split flow was enlarged up to 50 mL/min in the cleaning phase. We used a capillary column HP-5MS (30 m × 0.25 mm i.d., 0.25 μm film thicknesses) purchased from Agilent Technologies (Palo Alto, CA, USA) for the separation with an initial oven temperature of 100 °C (3 min) and posterior increment of 5 °C/min to 300 °C. The final temperature was held for 15 min. We used helium as the carrier gas at a constant flow rate of 0.8 mL/min. The temperature of the transfer line and the MS source were set at 300 °C and 240 °C, respectively.

For the chromatogram analyses, we used the software Xcalibur^TM^ 2.1.0.1140 (Thermo Fischer Scientific Inc., San Jose, CA, USA). Then, to identify the chemicals, we first compared their mass spectra in the NIST/EPA/NIH (NIST 02) computerised mass spectral library. Subsequently, we compared the spectra and retention times of compounds with commercial standards (from Sigma– Aldrich Chemical Co.) when these were available. No relevant impurities were found in the solvent and/or the control vial samples. We determined the relative amount of each compound using the percentage of the total ion current (TIC). We studied potential differences in the relative abundances of chemicals between C- and E-males by using general linear models (GLMs) analyses. In addition, once α-tocopherol was identified, its area in the chromatogram was relativized to the area of the internal standard (*i.e*., *n*-heptadecane), whose concentration was known, as we mentioned above. This procedure allowed confirmation of potential variations in relative abundance of VE regardless of the other components in the chemical secretions.

### Immune response

At the end of the dietary supplementation, we assessed the immune response of male lizards by conducting a delayed-type hypersensivity test (phytohemaglutinin injection test; PHA) by means of a subcutaneous injection of a mitogen (0.02 mg of PHA dissolved in 0.02 mL of phosphate-buffered saline, PBS) in the left hindlimb foot pad. We used a pressure-sensitive spessimeter to measure thickness (to the nearest 0.01 mm) at the point of injection before and 24 h after the injection at the marked point. For each point, we made five consecutive measurements and calculated an average value. Repeatability (see ref. 37) of these five measurements was very high (pre-injection: r = 0.9876, F_37,152_ = 269.30, *P* > 0.0001; post-injection: r = 0.9875, F_37,152_ = 274.00, *P* > 0.0001). Then, we calculated the lizard immune responses, subtracting pre- to post-injection average measures^38–41^. It has been suggested that physiological PHA reaction may be a nonspecific complex inflammation related to infiltration of cells, representing both adaptive and innate immunity^39, 42^. The final swelling may be result of a diverse index of cutaneous immune activity. Additionally, there is some debate on the meaning of a thicker swelling (see ref. 43). However, because of its simplicity, this test is often used in studies on lizards (e.g. refs 44–46). Therefore, with the PHA test, we aimed to assess a standardised index of immunocompetence, regardless of the types of immune cells concerned. To assess potential differences in immune responses between males of both treatments, we used a one-way analysis of variance (ANOVA) on log-transformed data.

### Chemosensory responses of females to scent of males

Tongue-flicking behaviour has been widely associated with squamate chemoreception^47^. Lizards and snakes extrude tongues to sample chemicals from the environment and other individuals^48, 49^. It is thought that differential TF rates are the result of stimuli discrimination. Thus, an increase in the elicited number of TF towards a given scent can be interpreted as a higher ‘interest’ in this particular scent^48^. At the end of the dietary supplementation, we measured the responses of females to scents (*i.e*., femoral secretions) from males in the two treatments (C-males and E-males). We compared the TF rates of females to swabs impregnated with the secretions of the two groups of males. These swabs were imbued with approximately the same amount of secretion (2 × 1 mm of waxy secretion from each of two femoral pores of a male), which avoided the problem of females responding to variation in the amount of secretion instead of to differences in chemical composition. We also used water as an odourless control stimulus. All of the females were exposed to the three stimuli (water *vs*. C-male *vs*. E-male) in a randomized order. The TF experiments were conducted in outdoor conditions during an interval of three days. We only performed one trial per day with each female to avoid stressing the lizards. Each female could bask at least 2 h before trials. We exposed the secretion-imbued swab to 2 cm anterior to the lizard´s snout. Then, we noted the number of TF directed to the swab along 1 min since the first TF. We also measured the latency, which is the time (s) that females required from the initial exposure of the swab to the first TF. We tested for potential differences in TF rates and latency of females using repeated measures ANOVAs with the type of scent (*i.e*., water *vs*. E-males vs. C-males) as a within factor. Differences between pairs of treatments were tested with post hoc Tukey’s tests.

All of the variables were previously log-transformed to ensure normality (Shapiro-Wilk’s test) as well as the homogeneity of variances (Levene’s test) in statistical analyses, which were conducted with R, version 3.3.1, and SPSS 20.0.0.
